# 5-Fluorouracil dose escalation generated desensitized colorectal cancer cells with reduced expression of protein methyltransferases and no epithelial-to-mesenchymal transition potential

**DOI:** 10.32604/or.2024.049173

**Published:** 2024-05-23

**Authors:** KIMBERLY FENECH, ISAAC MICALLEF, BYRON BARON

**Affiliations:** 1 Centre for Molecular Medicine and Biobanking, University of Malta, Msida, MSD 2080, Malta; 2 Department of Tumor Genetics and Biology, Graduate School of Medical Sciences, Faculty of Life Sciences, Kumamoto University, Kumamoto, 860-8556, Japan

**Keywords:** Chemoresistance, Epithelial-to-mesenchymal transition, Protein methylation, Protein methyltransferases

## Abstract

**Background:**

Colorectal cancer (CRC) is one of the most frequently diagnosed cancers. In many cases, the poor prognosis of advanced CRC is associated with resistance to treatment with chemotherapeutic drugs such as 5-Fluorouracil (5-FU). The epithelial-to-mesenchymal transition (EMT) and dysregulation in protein methylation are two mechanisms associated with chemoresistance in many cancers. This study looked into the effect of 5-FU dose escalation on EMT and protein methylation in CRC.

**Materials and Methods:**

HCT-116, Caco-2, and DLD-1 CRC cell lines were exposed to dose escalation treatment of 5-FU. The motility and invasive potentials of the cells before and after treatment with 5-FU were investigated through wound healing and invasion assays. This was followed by a Western blot which analyzed the protein expressions of the epithelial marker E-cadherin, mesenchymal marker vimentin, and the EMT transcription factor (EMT-TF), the snail family transcriptional repressor 1 (Snail) in the parental and desensitized cells. Western blotting was also conducted to study the protein expressions of the protein methyltransferases (PMTs), Euchromatic histone lysine methyltransferase 2 (EHMT2/G9A), protein arginine methyltransferase (PRMT5), and SET domain containing 7/9 (SETD7/9) along with the global lysine and arginine methylation profiles.

**Results:**

The dose escalation method generated 5-FU desensitized CRC cells with distinct morphological features and increased tolerance to high doses of 5-FU. The 5-FU desensitized cells experienced a decrease in migration and invasion when compared to the parental cells. This was reflected in the observed reduction in E-cadherin, vimentin, and Snail in the desensitized cell lines. Additionally, the protein expressions of EHMT2/G9A, PRMT5, and SETD7/9 also decreased in the desensitized cells and global protein lysine and arginine methylation became dysregulated with 5-FU treatment.

**Conclusion:**

This study showed that continuous, dose-escalation treatment of 5-FU in CRC cells generated 5-FU desensitized cancer cells that seemed to be less aggressive than parental cells.

## Introduction

Despite recent therapeutic advancements, cancer remains among the leading causes of worldwide mortality, accounting for 10 million deaths in 2020 [1]. Also, for 2020, CRC was classified as the third most diagnosed cancer and the second leading cause of cancer-related deaths [2]. The five-year survival rate of CRC varies from 90% for patients diagnosed at stage I to 10% for patients diagnosed with metastatic disease [3].

Advanced CRCs present poor prognosis primarily due to chemotherapy failure [1]. Indeed, cancer chemotherapy becomes ineffective once chemoresistance sets in. Chemoresistance refers to the innate or acquired ability of cancer cells to evade or survive the administered therapeutics such as 5-FU based chemotherapeutic agents [4]. This contributes to cancer relapse and progression, treatment failure, and eventually the patient’s death [5].

There are several mechanisms associated with chemoresistance, one of which is EMT. It is orchestrated through the expression of EMT-TFs, including Snail, snail family transcriptional repressor 2 (Slug), and twist family BHLH transcription factor 1 (Twist 1). EMT-TFs suppress the expression of epithelial markers such as E-cadherin [6,7]. The reduction of E-cadherin expression is one prominent feature of EMT and has been associated with a poor prognosis when found in Stage III tumors of CRC. Moreover, EMT-TFs induce the gene expression of mesenchymal markers including vimentin [7]. The loss of epithelial gene expression and induction of mesenchymal marker expression causes a shift from an apical-basal polarity to a front-rear polarity, loss of epithelial shape, and the disruption of cell-to-cell junctions, supporting cell migration and invasion [8,9]. Several drug-resistant cancers have been shown to present such an EMT phenotype and this has been further linked to cancer metastasis, enhanced expression of genes related to drug resistance, as well as a cancer stem cell (CSC) phenotype [5,10]. For instance, it was shown that Snail and Slug overexpression in non-small cell lung cancer (NSCLC) cells promoted gefitinib resistance [11]. Additionally, the induction of EMT in breast cancer cells enhanced the levels of ATP-binding cassettes (ABC) transporters [12]. Furthermore, the overexpression of Twist in NSCLC cell lines brought about suppression in the pro-apoptotic marker, Bcl-2 Interacting Mediator of cell death (BIM), resulting in decreased apoptosis after treatment with gefitinib [13]. Finally, chemoresistance towards 5-FU in CRC cells was associated with reduced protein levels of E-cadherin and higher protein expression levels of fibronectin (a mesenchymal marker) and EMT-TF’s, Twist1, Zinc-finger E-box-binding homeobox 1 (Zeb1) and Zinc-finger E-box-binding homeobox 2 (Zeb2) [14].

Scientists have also linked post-translational modifications (PTMs) particularly, protein methylation, as another mechanism involved in drug resistance. Protein methylation occurs on both histone and non-histone proteins, with the main amino acid residues altered being lysine and arginine. It is catalyzed by protein lysine methyltransferases (PKMTs) or protein arginine methyltransferases (PRMTs) depending on which residue is methylated [15].

Numerous studies have shown that PMTs contribute to the progression of EMT through the regulation of the gene expression of several EMT markers. For example, in breast, ovarian, and lung cancers, inhibition of the PKMT, EHMT2 reduced the metastatic potential of the cells as the expression of N-cadherin, vimentin, Snail and Slug were downregulated, while the E-cadherin promoter activity was enhanced [16–18]. Similarly, in head and neck squamous cell carcinoma (HNSCC), the overexpression of the arginine methyltransferase PRMT5 resulted in increased Twist1 and N-cadherin levels, whilst E-cadherin levels decreased [19]. Additionally, the downregulation of PRMT5 gene expression in CRC cells, reduced the mRNA expression of vimentin, β-catenin, and collagen I, once again showing the involvement of PRMT5 in EMT [20].

Conversely, the role of the PKMT, SETD7/9 is contradictory in cancer, with some studies showing it has a tumor suppressive role and others showing its importance in cancer-related processes and drug resistance [21,22]. In particular, SETD7/9 was shown to inhibit EMT in breast cancer and hepatocellular carcinoma whereby its overexpression promoted the upregulation of E-cadherin expression and downregulated vimentin [21].

While it is widely recognized that dysregulation in protein methylation contributes to cancer formation and progression, further research is essential to identify driver methylation targets in a cancer-specific context that can be used for diagnosis, screening, monitoring, and prognosis purposes [23]. This study aims to uncover the roles of PMTs in EMT in CRC to explore whether these PMTs contribute to a poorer prognosis in CRC. Additionally, this study investigates how chemotherapeutic inhibitory strategies impact protein methylation in CRC cells and influence their EMT potential, providing insights into how the cells are responding to treatment. In this study, the high-level laboratory model of drug resistance was employed. Here, the CRC cells were exposed to dose-escalating, continuous 5-FU treatment [24]. This simplified the identification of those molecular changes brought about by drug resistance in a relatively shorter amount of time [24]. Indeed, the influence of dose escalation 5-FU treatment in CRC cells was investigated on their viability and metastatic potential. In addition, the protein expression of the EMT markers Snail, E-cadherin, and vimentin, the PMTs EHMT2, PRMT5, and SETD7/9, as well as the overall protein lysine and arginine methylation levels were compared between the parental and their 5-FU desensitized equivalent CRC cells.

## Materials and Methods

### Reagents and materials

HCT-116 (CCL-247), Cancer coli-2 (Caco-2) (HTB-37), and DLD-1 (CCL-221) cell lines were purchased from the American Type Culture Collection (ATCC^®^), Virginia, USA. The following compounds were purchased from Sigma-Aldrich, Chemie GmbH, Germany; Dulbecco’s Modified Eagle Medium (DMEM) F-12, ethylenediaminetetraacetic acid (EDTA), 5-FU and Bovine serum albumin (BSA). Penicillin-streptomycin (pen-strep) and Amphotericin B were purchased from Gibco, Waltham, Massachusetts, USA. Foetal bovine serum (FBS) was acquired from Nichirei BioSciences, Tokyo, Japan. PrestoBlue^®^ reagent was purchased for Invitrogen, ThermoScientific, Massachusetts, USA. The two well cell culture inserts for the wound healing assays were acquired from Ibidi, Gräfelfing, Germany, and the transwell inserts from cellQart^®^, SABEU GmbH & Co. KG, Northeim, Germany. The Quick StartTM Bradford protein assay was purchased from Bio-Rad, California, USA. E-cadherin mouse monoclonal antibody was purchased from R&D systems, Bio-Techne, Abington England, Snail rabbit monoclonal antibody from Novus Biologicals, Bio-Techne, Abington, England, and vimentin mouse monoclonal antibody from SantaCruz, Biotechnology, Heidelberg, Germany. EHMT2 rabbit monoclonal antibody, PRMT5 mouse monoclonal antibody, SET7/9 rabbit monoclonal antibody, mono-methyl lysine rabbit monoclonal antibody, di-methyl lysine rabbit monoclonal antibody, tri-methyl lysine rabbit monoclonal antibody, mono-methyl arginine monoclonal antibody, symmetric dimethyl arginine monoclonal antibody and asymmetric dimethyl arginine monoclonal antibody were acquired from Cell Signalling Technology^®^ (CST), Leiden, The Netherlands. GAPDH mouse monoclonal antibody was purchased from ProteinTech, Manchester, UK. Amersham ECL Mouse IgG HRP-linked whole antibody and Amersham ECL rabbit IgG HRP-linked whole antibody were obtained from Li-Cor^®^ BioSciences, Nebraska, USA. Immunostar^®^ LD (Wako) detection reagent was obtained from GE Healthcare, Chicago, USA. All other reagents used were available in the laboratory.

### Cell culture

Three CRC cell lines, namely HCT-116, Caco-2, and DLD-1 cells were investigated in this study. Cells were cultured as parental or 5-FU desensitized cells. They were maintained at 37°C, 5% CO_2_, and 95% humidity in DMEM F-12 completed with 10% FBS, 1% pen-strep and 1% Amphotericin B. Alternatively, cells were cultured using DMEM F-12 supplemented with 10% human plasma 1% pen-strep, 1% Amphotericin B, 0.6% of 0.2 mg/mL platelet lysate, and 0.1% of 60 mg/0.6 mL heparin (commercially available as Clexane^®^) as described by Zammit et al. [25].

### Drug treatment

**Figure 1:**
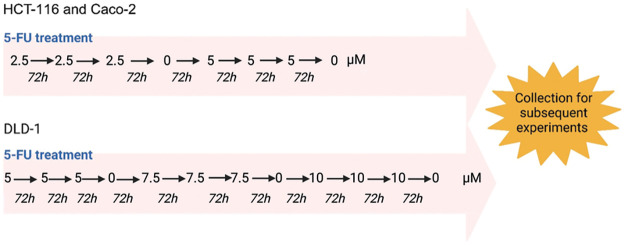
The dose escalation method used for the 5-FU treatment of CRC cells. HCT-116, Caco-2 and DLD-1 cells were exposed to increasing doses of 5-FU. The HCT-116 and Caco-2 were exposed to a range of 2.5 to 5 µM while the DLD-1 cells were desensitized with a range of 5 to 10 µM. Created using Biorender.com.

The high-level laboratory model of drug resistance described by Dermot et al. makes use of dose escalation drug treatment [24]. In this study, dose escalation (Fig. 1) was initiated from 2.5 µM of 5-FU added to T-25 flasks of HCT-116 and Caco-2 cells [26]. 5-FU treatment of DLD-1 cells was initiated from 5 µM of 5-FU [26]. Cells were monitored for morphological and confluency changes every three days. Once cell culture presented a high confluency after three days, another dose of 5-FU was administered. If not, cells were untreated for a period of time to allow for recovery. A particular dose of 5-FU was administered consecutively three times, after which this process was repeated with a higher dose of 5-FU. The HCT-116 and Caco-2 cells were desensitized up to 5 µM while the DLD-1 cells were desensitized up to 10 µM of 5-FU. The desensitized cells were maintained in the presence of the drug until they were collected for subsequent experiments. Prior to collection, cells were cultured in drug-free medium for a duration of 3–7 days.

### Cell viability assay

Parental and 5-FU desensitized cells were seeded in 96-well plates (5.0 × 10^3^ cells/well) with complete medium and were left to adhere for 24 h. Following attachment, parental and desensitized HCT-116 and Caco-2 cells were desensitized with 10 µM of 5-FU, diluted in complete medium [26]. Parental and 5-FU exposed DLD-1 cells were desensitized with 20 µM of 5-FU [26]. At each place, certain wells for both parental and desensitized cells were exposed to 20 mM of hydrogen peroxide (H_2_O_2_) which acted as a cytotoxic agent by promoting oxidative stress [26]. These wells served as a positive control and were used to blank the absorbance data [26]. The plates were incubated at 37°C, 5% CO_2_, and 95% humidity for 72 h [26]. Then, the medium in each well was replaced with 10% PrestoBlue^®^ reagent diluted in DMEM F-12 [26]. Absorbance readings were taken after 2 h using the Mithras LB940 microplate reader at 540 nm [26].

### Wound healing assay

Wound healing assays were performed in two-well cell culture inserts fitted into 24-well plates [26]. Parental and 5-FU exposed CRC cells were seeded in the silicone cell culture inserts at a high density. A 500 μL aliquot of complete medium was pipetted around each insert [26]. Cells were left to adhere for 24 h at 37°C, 5% CO_2_, and 95% humidity. After adherence, the cell culture inserts were carefully removed. Bright field images of the cells were taken at 0 and 72 h on a Nikon Eclipse Ti microscope [26]. T-scratch software was used to generate the percentage (%) open wound areas for each image from which migration potential was calculated [27].

### Invasion assays

Parental and 5-FU desensitized CRC cells were seeded at a high cell density on the upper surface of the transwell insert chamber which contained pore sizes of 8 μm [26]. The well was filled with 500 μL of complete DMEM F-12, beneath the transwell insert [26]. The plate was incubated for 72 h at 37°C, 5% CO_2_, and 95% humidity, allowing the cells to trans-migrate to the bottom chamber, containing fresh, complete medium [26]. Afterward, the well surface was imaged at a magnification of ×100 [26]. The cell counter plug-in of ImageJ was used to count the cells that successfully managed to invade the membrane [28].

### Western blotting

Parental and desensitized CRC cells were pelleted and then lysed with urea lysis buffer (composed of 8 M urea, 1.5 M thiourea, 0.5 M NaCl). Bradford reagent was used to determine protein concentration on an Eppendorf Bio Photometer Plus UV/Vis spectrophotometer. SDS-PAGE (10% resolving and 6% stacking gels) was carried out using the Atto AE-6450 Dual Mini Slab Electrophoresis Kit [26]. The amount of protein loaded per lane was 20 μg. Electrophoresis was carried out at constant current 20 mA and 300 V for 50–60 min. Wet electroblotting was carried out using the Mini Trans-Blot Cell^®^ Module (Bio-Rad) set at 100 V and 350 mA for 65 min. Following electroblotting, the membrane was stained with 0.1% Ponceau S solution and then blocked with 5% skimmed milk for 1 h [26]. The membrane was then incubated overnight at 4°C with primary antibodies diluted in 5% BSA solution at a ratio of 1:250 to 1:50,000 [26]. The following day, the membrane was incubated for 1 h with the secondary antibody diluted in 5% BSA at a ratio of 1:5000. The primary and secondary antibodies used targeted EHMT2 (1:250), PRMT5 (1:500), SETD7/9 (1:250), mono-methylated lysine (MMK), di-methylated lysine (DMK), tri-methylated lysine (TMK), mono-methylated arginine (MMA) (1:1000), symmetric dimethylated arginine (SDMA) (1:1000), asymmetric dimethylated arginine (ADMA) (1:1000), E-cadherin (1:500), vimentin (1:500), Snail (1:500) and GAPDH (1:50,000). Visualization of bands was carried out using the ChemiDoc Imaging system. The bands of the proteins of interest were relatively quantified against GAPDH using ImageJ [26].

### Statistical analysis

Statistics on the cell viability, migration, and invasion assay data were performed using IBM SPSS software (version 26) [26]. First, the Shapiro Wilk normality test was applied to the normalized data [26]. Levene’s test followed by an independent sample *t*-test was carried out on the data of the viability and invasion assay, since the data was normally distributed [26]. The Mann Whitney U-test was used for the data of the wound healing assay since the data was not normally distributed [26]. Statistical significance was depicted by * for *p* < 0.05 and ** for *p* < 0.01. *p*-values larger than 0.05 were not considered statistically significant.

## Results

### Morphology observations of parental and 5-FU desensitized CRC cells

**Figure 2:**
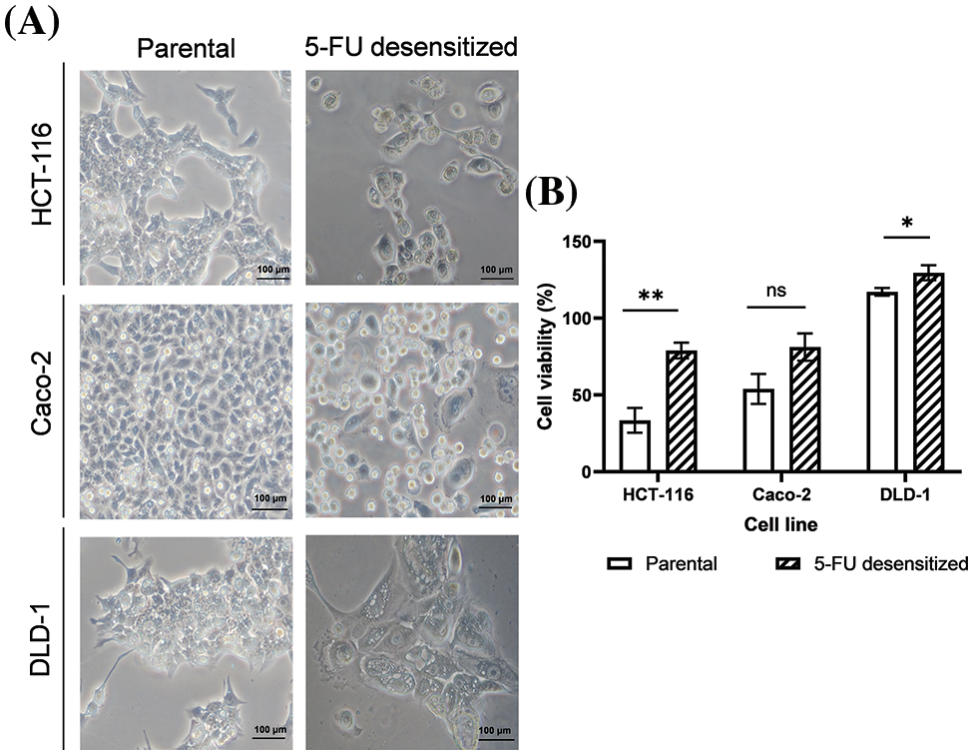
Dose escalation treatment of 5-FU in CRC cells led to morphological changes and enhanced tolerance to higher doses of 5-FU. (A) Microscopy images of parental and desensitized HCT-116, Caco-2 and DLD-1 cells. Scale bar: 100 µM. (B) The percentage average cell viability of parental and 5-FU desensitized CRC cells after 72 h (n = 6). Parental and desensitized HCT-116 (exposed to 2.5–5 µM of 5-FU in culture) were desensitized with a single dose of 10 µM of 5-FU. Parental and desensitized DLD-1 cells (exposed to 5–10 µM of 5-FU in culture) were desensitized with 20 µM of 5-FU. Result is presented as average ± SEM of three technical repeats across two biological repeats for each cell line. Significance is presented as *p < 0.05, **p < 0.001 and ns non-significant as determined from independent sample *t*-test.

5-FU treatment was carried out using the dose-escalation method whereby the CRC cells were exposed to increasing doses of 5-FU [24,26]. The HCT-116 and Caco-2 cells were exposed to a range of 2.5–5 μM of 5-FU while the DLD-1 cells were exposed to a range of 5–10 μM of 5-FU. All cell lines (parental and 5-FU desensitized) were cultured as adherent cells [26], with the 5-FU desensitized cells presenting a number of morphological differences from their parental counterparts (Fig. 2A). In general, with continuous 5-FU treatment, all cell lines presented cells that became larger in size. The 5-FU desensitized HCT-116 and Caco-2 cells were characterized by an irregular cell shape and numerous vacuoles, with some cells presenting enlarged nuclei or multinucleation. Such morphological changes have been observed following treatment with a variety of chemotherapeutic drugs in a number of cancers and are associated with chemoresistance [29].

### Cell viability of parental and 5-FU desensitized CRC cells

Parental and 5-FU desensitized HCT-116, Caco-2, and DLD-1 cells were treated with double the concentration of 5-FU that was used in culture, to assess their tolerance to the drug in relation to cell viability and investigate if the observed morphological changes in the desensitized cells were indeed associated with increased resistance to 5-FU treatment (Fig. 2B). All 5-FU desensitized cells showed a higher percentage cell viability than their corresponding parental cells when desensitized with the same dose of the drug. Specifically, the parental HCT-116 cells exhibited a 46% higher cell death than the 5-FU desensitized HCT-116 cells (*p*-value of 0.001). Likewise, compared to their respective 5-FU desensitized cells, the parental Caco-2 cells (*p*-value of 0.065) presented a 27% increase in cell death while the parental DLD-1 cells had a 12% increase in cell death (*p*-value of 0.046). All results were statistically significant except for the Caco-2 cell line, however the data trended towards significance.

### Cell migration and invasion in parental and 5-FU desensitized CRC cells

**Figure 3:**
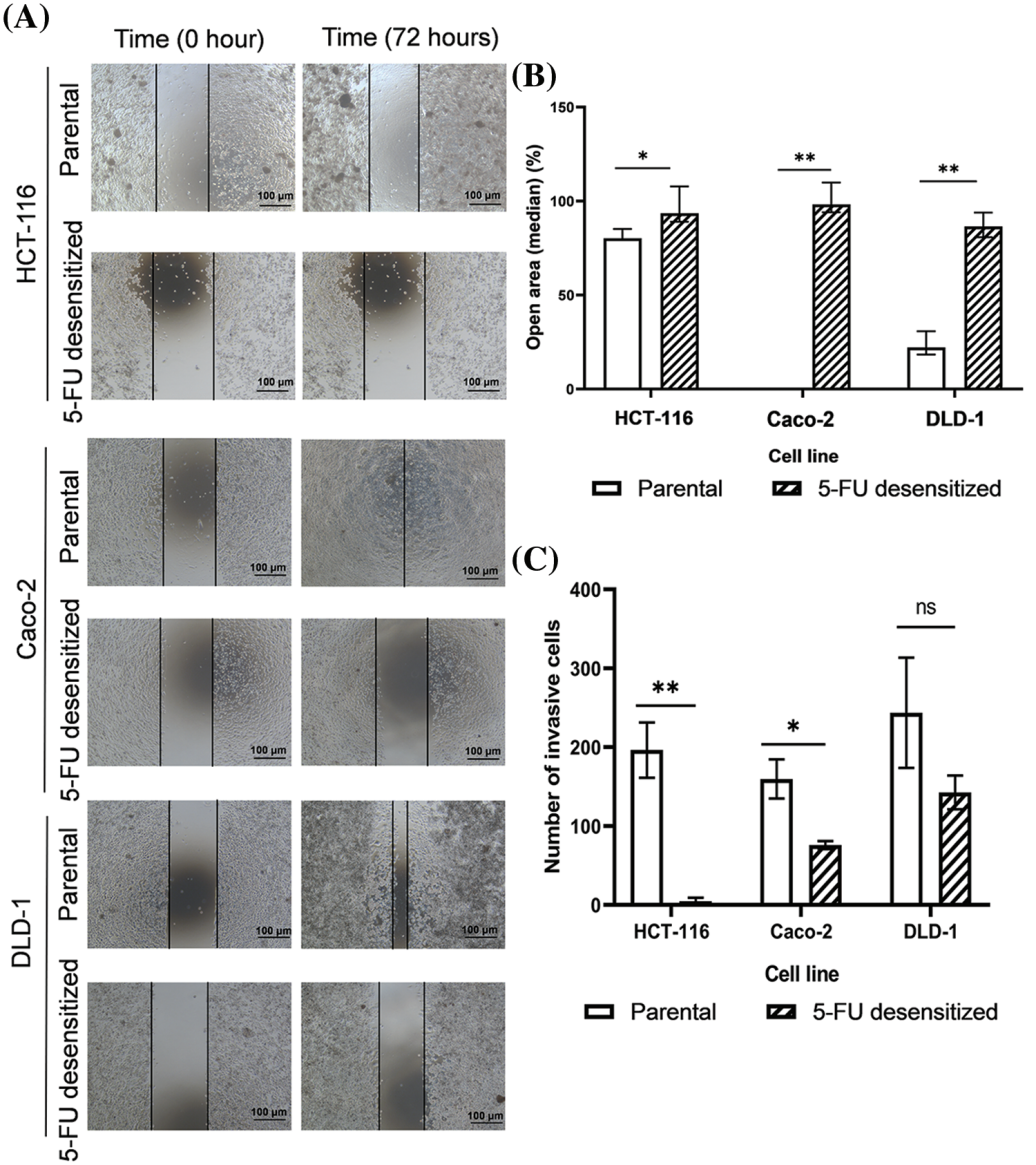
Dose escalation treatment of 5-FU decreased the migration and invasion abilities of CRC cells. (A) Microscopy images of wound healing assays of parental and 5-FU desensitized HCT-116, Caco-2 and DLD-1 cells for one technical replicate across one biological repeat. Scale bar: 100 µM. (B) The median % open areas of parental and 5-FU desensitized cells after 72 h (n = 6). Result is presented as median ± upper and lower quartiles of three technical repeats across two biological repeats for each cell line. Significance is presented as *p < 0.05 and **p < 0.001 as determined from the Mann Whitney U-test. (C) The number of invasive cells for parental and 5-FU desensitized HCT-116, Caco-2 and DLD-1 cells (n = 3). Result is presented as average ± SEM of three technical repeats for each cell line. Significance is presented as *p < 0.05 and **p < 0.001 and ns non-significant as determined from the independent sample *t*-test.

Wound healing and invasion assays were performed to compare the metastatic potential of parental and 5-FU desensitized CRC cells, as both migration and invasion serve as indicative behavioral markers for metastasis (Fig. 3). In this study, all 5-FU desensitized cells had weaker migratory and invasive abilities than their parental counterparts.

Considering the wound healing assay, the percentage open areas of 5-FU desensitized cells for the HCT-116 (*p*-value of 0.016), Caco-2 (*p*-value of 0.003), and DLD-1 (*p*-value of 0.004) were significantly higher than the parental cells after 72 h (Figs. 3A and 3B). In contrast to the 5-FU desensitized cells, the parental HCT-116 showed a 12% higher wound area closure after 72 h. The most significant difference in cell motility was noted between the parental and 5-FU desensitized Caco-2 cells, with the parental cells showing a 98% increase in wound area closure. Similarly, the wound area of the parental DLD-1 cells showed a 64% greater closure than the parental cells.

Moreover, the number of invading parental HCT-116 and Caco-2 cells were 95% (*p*-value of 0.006) and 94% (*p*-value of 0.03) higher respectively, in comparison to their corresponding 5-FU-desensitized counterparts (Fig. 3C). Likewise, the parental DLD-1 cells were 84% more invasive than the 5-FU desensitized DLD-1 cells (*p*-value of 0.239), however, this difference was not statistically significant (Fig. 3C). Hence, in this study, long-term 5-FU exposure was not accompanied by an increase in the metastatic potential of the CRC cells.

### Protein analysis of EMT markers in parental and 5-FU desensitized CRC cells

**Figure 4:**
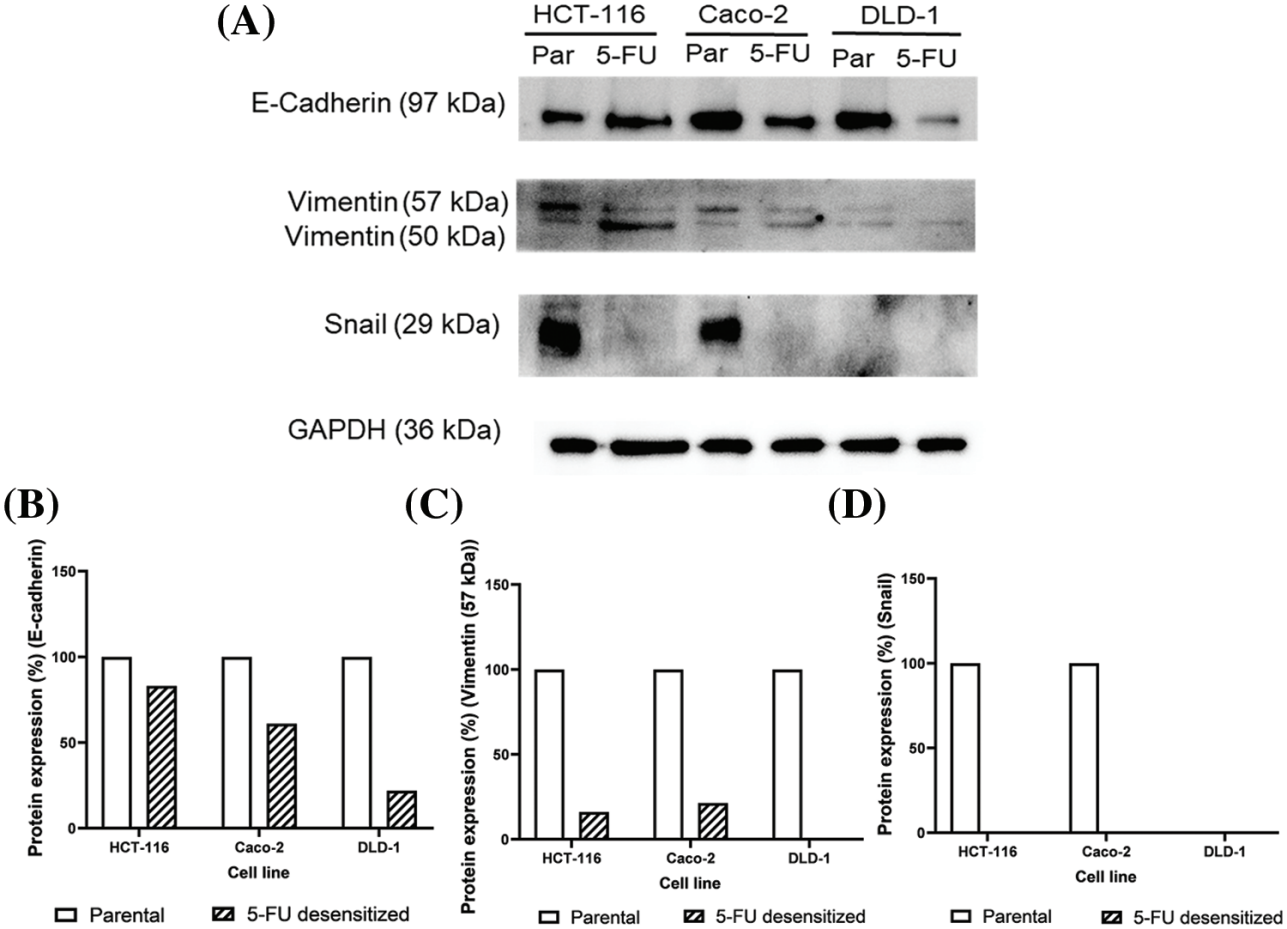
Dose escalation exposure of 5-FU promoted the downregulation of key EMT markers. (A) Western blots showing the decrease in protein levels of E-cadherin, vimentin and Snail in 5-FU desensitized (5-FU) cells when compared to parental (par) HCT-116, Caco-2 and DLD-1 cells. (B–D) The quantification of % protein expression of E-cadherin, vimentin (57 kDa) and Snail in both cell types across one technical and biological repeat. The 57 kDa form of vimentin was only quantified as the 50 kDa is a truncated form of the protein.

To validate the observed loss in migration and invasion potential in the desensitized cells, the protein levels of the epithelial marker, E-cadherin, the mesenchymal marker vimentin, and the EMT-TF Snail were analyzed in parental and 5-FU desensitized HCT-116, Caco-2, and DLD-1 cells via Western blotting to validate the observed loss in migration and invasion in the desensitized cells (Fig. 4). Additionally, this analysis aimed to investigate whether EMT was one of the mechanisms involved in the acquisition of 5-FU resistance. The protein expression of E-cadherin was lower in all 5-FU desensitized cells (Fig. 4A). Specifically, E-cadherin decreased by 17% in the desensitized HCT-116 cells and by 39% in the desensitized Caco-2 cells (Fig. 4B). Likewise, 5-FU treatment in the DLD-1 cells induced the reduction of E-cadherin by 78% (Fig. 4B). Vimentin generated two bands at molecular weights of 50 and 57 kDa. Here, the 57 kDa protein band was quantified as this represents the intact form of vimentin [30]. Compared to the parental cells, an 84% and 79% reduction in vimentin was observed in the desensitized HCT-116 and Caco-2 cells, respectively (Fig. 4C). The 57 kDa isoform of the protein was not detected in the 5-FU desensitized DLD-1 cells (Fig. 4C). Lastly, Snail was not observed in the parental DLD-1 cells and in the three desensitized cell lines (Fig. 4D). Snail was only detected in the parental HCT-116 and Caco-2 cells. Taken together, long-term 5-FU treatment resulted in the loss of protein expression of the three EMT markers across the three cell contexts.

### Protein analysis of EHMT2, PRMT5 and SETD7/9 in parental and 5-FU desensitized CRC cells

**Figure 5:**
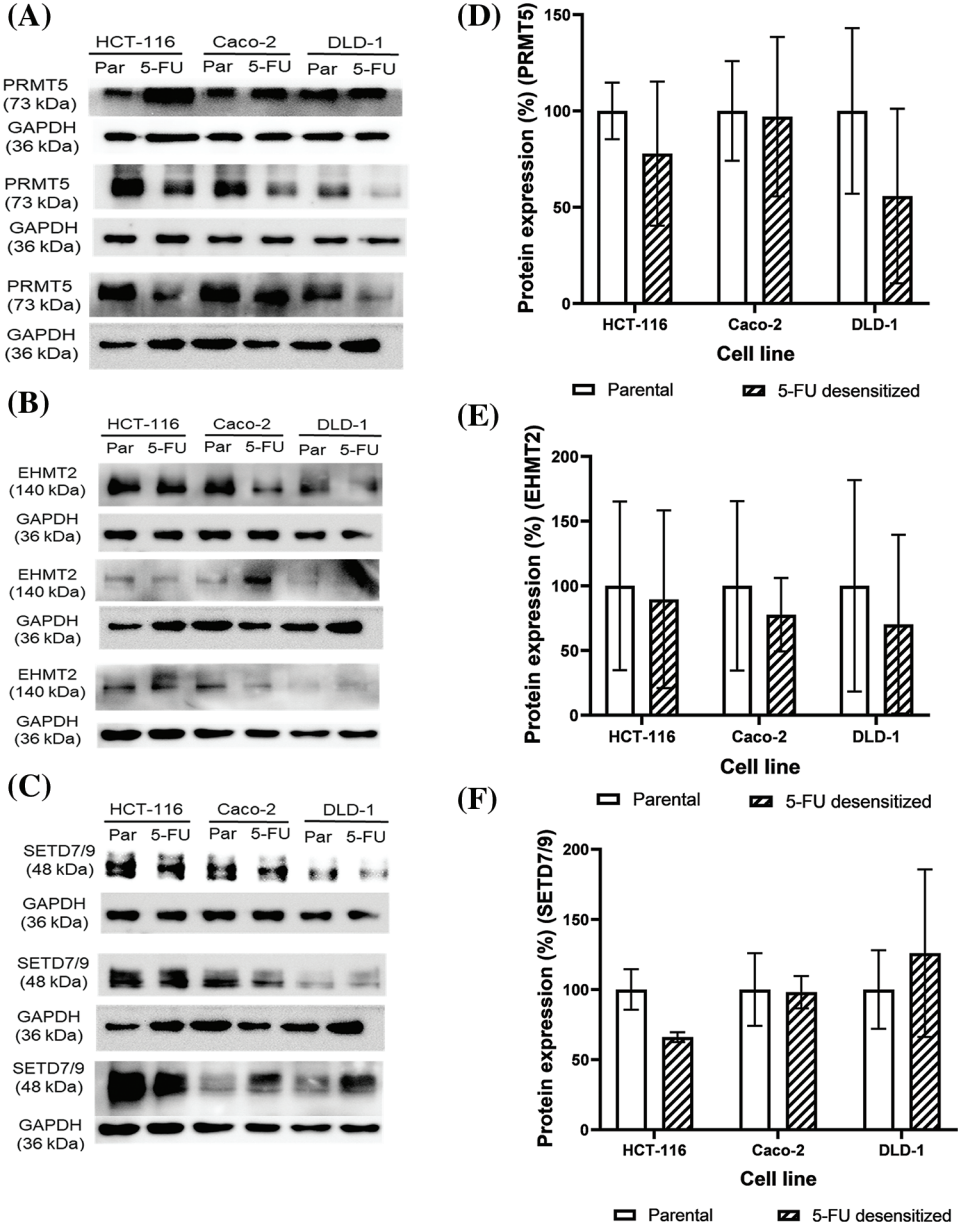
Dose escalation exposure of 5-FU promoted the dysregulation of PMT protein expressions in CRC cells. (A–C) Western blots of EHMT2, PRMT5 and SETD7/9 in the parental (par) and desensitized (5-FU) cells, respectively. (D–F) Quantification of the % protein expression of EHMT2, PRMT5 and SETD7/9 in parental and 5-FU desensitized cells of one technical replicate across three biological repeats.

Western blotting was carried out to study how 5-FU treatment affected the protein expression of EHMT2, PRMT5, and SETD7/9 and to investigate whether the loss in EMT affected the expression of such PMTs. Additionally, protein analyses also investigated the difference in the expression of the three PMTs between the parental and 5-FU desensitized HCT-116, Caco-2, and DLD-1 cells (Figs. 5A–5C).

With continuous 5-FU treatment, PRMT5 protein expression decreased by 22% in all the three biological repeats of the HCT-116 cells (Fig. 5D). Likewise, PRMT5 levels were lower in two of three biological repeats of the 5-FU desensitized Caco-2 cells in comparison to the parental cells (Fig. 5A). Quantification of PRMT5 across the three repeats showed that its expression decreased by an average of only 3% in the 5-FU desensitized Caco-2 cells (Fig. 5D). Then, PRMT5 levels decreased by an average of 44% across the three biological repeats of the 5-FU desensitized DLD-1 cells when compared to the parental cells (Fig. 5D). In comparison to the parental HCT-116 cells, EHMT2 (isoform S at 140 kDa) protein expression decreased by 10% across the three biological repeats of the 5-FU desensitized cells (Fig. 5E). Similarly, a reduction in EHMT2 levels were observed in the 5-FU desensitized Caco-2 cells investigated in two of three biological repeats (Fig. 5B). Quantification of EHMT2 across all the biological repeats of the 5-FU desensitized Caco-2 cells revealed that EHMT2 decreased by 22% (Fig. 5E). Lastly, across the three biological repeats, 5-FU desensitized DLD-1 cells experienced an average of 30% loss in EHMT2 levels (Fig. 5E). SETD7/9 protein expression levels decreased by an average of 34% with continuous, dose-escalation 5-FU treatment of HCT-116 cells (Fig. 5F). Once again, in contrast to the parental Caco-2 cells, SETD7/9 levels decreased in two of three biological repeats of the 5-FU desensitized cells (Fig. 5C). Considering all biological repeats of the Caco-2 cells, SETD7/9 protein levels decreased by only 2% with repeated exposure of 5-FU (Fig. 5F). Finally, in comparison to parental cells, SETD7/9 protein levels increased in one biological repeat of the desensitized DLD-1 cells (Fig. 5C). In the second biological repeat, there was no difference in the expression of SETD7/9 between the two cell groups and in the third, a loss in the protein levels of SETD7/9 was observed in the 5-FU desensitized DLD-1 cells (Fig. 5C). Taken together, with dose-escalation 5-FU treatment, quantitative analysis showed that the SETD7/9 increased by an average of 26% in the DLD-1 cells (Fig. 5F). Therefore, in general, long term 5-FU exposure promoted a decrease in the protein levels of EHMT2, PRMT5 and SETD7/9 in the three CRC cell lines.

### Lysine methylation in parental and 5-FU desensitized CRC cells

**Figure 6:**
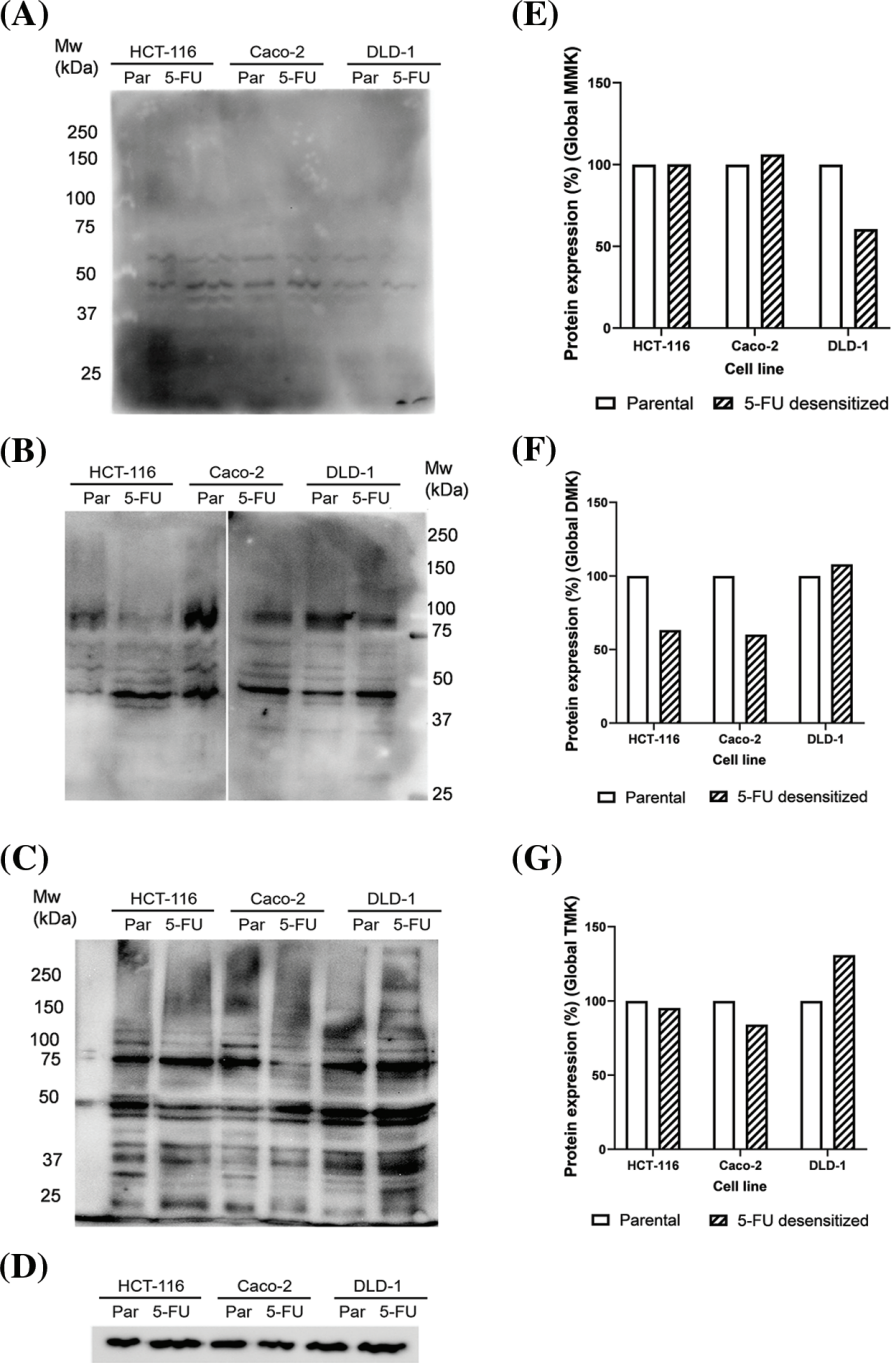
Dose escalation exposure of 5-FU promoted changes in global lysine methylation in CRC cells. (A) Western blot of MMK profile in parental (par) and 5-FU desensitized (5-FU) HCT-116, Caco-2 and DLD-1 cells. (B) Western blot of DMK profile in parental (par) and 5-FU desensitized (5-FU) HCT-116, Caco-2 and DLD-1 cells. (C) Western blot of TMK profile in parental (par) and 5-FU desensitized (5-FU) HCT-116, Caco-2 and DLD-1 cells. (D) Western blot of GAPDH loading control. (E–G) Quantification of % protein expression of global MMK, DMK and TMK residues in parental and 5-FU desensitized HCT-116, Caco-2 and DLD-1 cells across one technical and biological repeat.

**Figure 7:**
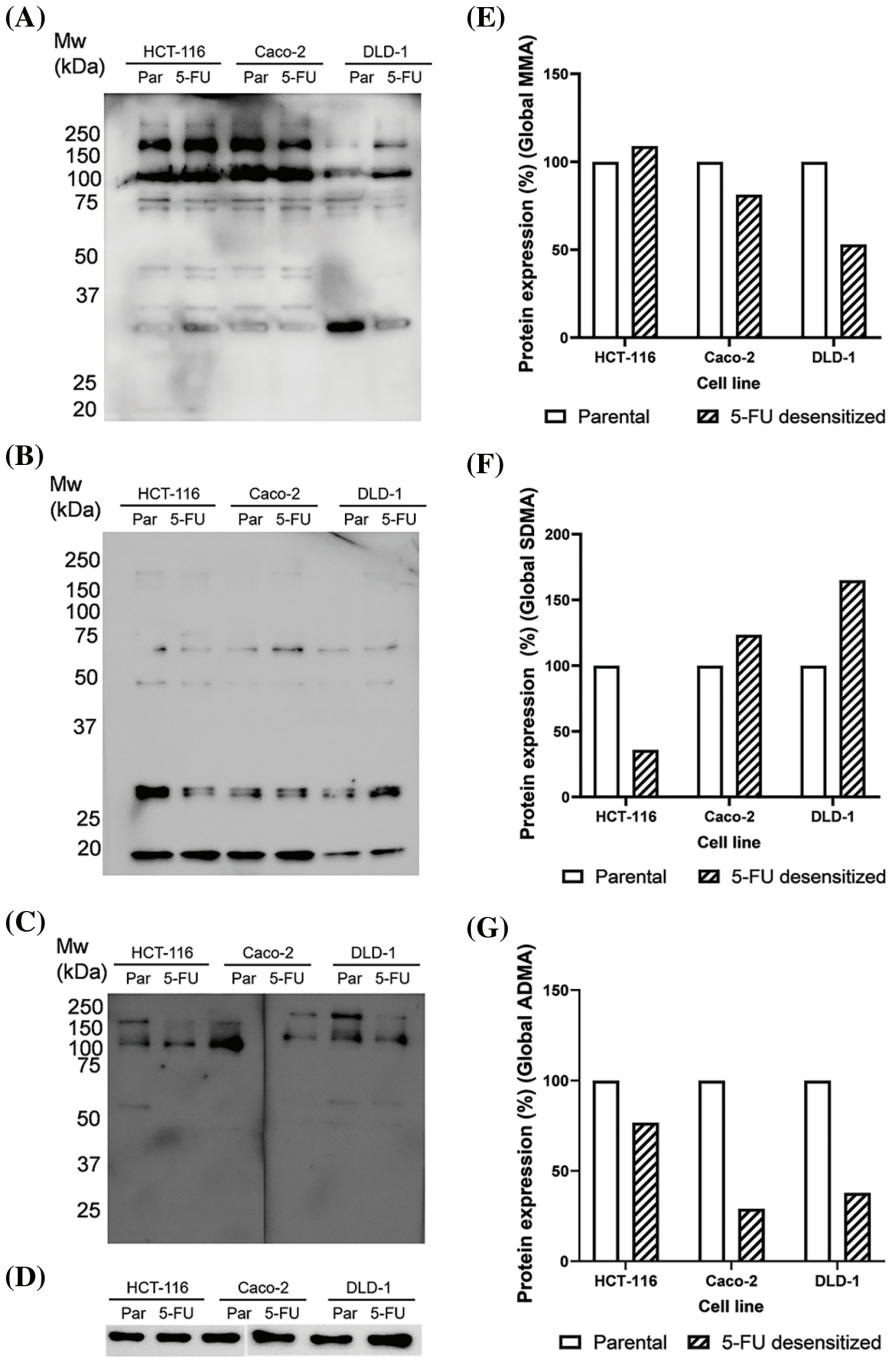
Dose escalation exposure of 5-FU promoted changes in global arginine methylation in CRC cells. (A) Western blot of MMA profile in parental (par) and 5-FU desensitized (5-FU) HCT-116, Caco-2 and DLD-1 cells. (B) Western blot of SDMA profile in parental (par) and 5-FU desensitized (5-FU) HCT-116, Caco-2 and DLD-1 cells. (C) Western blot of ADMA profile in parental (par) and 5-FU desensitized (5-FU) HCT-116, Caco-2 and DLD-1 cells. (D) Western blot of GAPDH loading control. (E–G) Quantification of % protein expression of global MMA, SDMA and ADMA residues in parental and 5-FU desensitized HCT-116, Caco-2 and DLD-1 cells across one technical and biological repeat.

Western blotting was also carried out to analyze how long-term 5-FU treatment affected the protein methylation profile of the cells. Global lysine (Figs. 6A–6C) and arginine (Figs. 7A–7D) methylation patterns were characterized for parental and 5-FU desensitized HCT-116, Caco-2, and DLD-1 cells. The analysis involved determining the percentage quantification of global lysine and arginine methylation in the desensitized cells in comparison to that in the parental cells. This was carried out to understand the influence of 5-FU treatment on protein methylation in CRC cells. Methylated protein bands detected throughout the molecular weight range represented several proteins presenting methylated lysine or arginine residues which overlap as a result of only small differences in molecular mass. Global lysine methylation involves the investigation of mono-, di- and tri-methylated lysine residues. Parental and 5-FU desensitized HCT-116 cells presented similar global MMK methylation (Fig. 6E). The 5-FU desensitized Caco-2 cells presented only a 6% increase in global MMK methylation compared to their parental counterparts (Fig. 6E). Conversely, the 5-FU desensitized DLD-1 cells presented a 43% decrease in global MMK methylation compared to their parental counterparts (Fig. 6E). Then, the protein expression levels of global DMK methylation were 37% and 40% lower in the 5-FU desensitized HCT-116 and Caco-2 cells when compared to their respective parental counterparts (Fig. 6F). An overall 8% increase in global DMK methylation was observed in the 5-FU desensitized DLD-1 cells when compared to the parental cells (Fig. 6F). The 5-FU desensitized HCT-116 cells showed only a small reduction (5%) in global TMK methylation in comparison to the parental cells (Fig. 6G). Similarly, the 5-FU desensitized Caco-2 cells experienced a loss of 16% in global TMK methylation when compared to their parental counterparts. In contrast, global TMK methylation increased by 31% in the desensitized DLD-1 cells (Fig. 6G). In summary, Fig. 6 shows a greater number of bands in the DMK and TMK profiles than in the MMK profile for all parental and 5-FU desensitized cell groups. Therefore, many proteins in the parental and 5-FU desensitized CRC cells, contain higher amounts of di- or tri-methylated lysine residues than mono-methylated lysine residues.

### Arginine methylation in parental and 5-FU desensitized CRC cells

Arginine methylation included the characterization of mono-methylated (MMA), symmetrically di-methylated (SDMA), and asymmetrical di-methylated (ADMA) residues. A minor increase (8%) in global MMA methylation was observed in the HCT-116 cells as they were subjected to escalating doses of 5-FU. Then, the 5-FU desensitized Caco-2 cells showed a 19% reduction in global MMA methylation in comparison to their parental counterparts (Fig. 7E). Likewise, the drug-exposed DLD-1 cells showed a reduction of 46% in global MMA methylation than those in the parental cells (Fig. 7E). Global SDMA methylation decreased by 64% in the 5-FU desensitized HCT-116 cells in comparison to their parental counterparts (Fig. 7F). Conversely, higher global SDMA methylation was detected in 5-FU desensitized Caco-2 and DLD-1 cells by 23% and 65%, respectively (Fig. 7F).

Global ADMA methylation levels decreased by 23% and 71% respectively as the HCT-116 and Caco-2 cells were exposed to increasing doses of 5-FU (Fig. 7G). Finally, there was a 62% reduction in global ADMA methylation in 5-FU desensitized DLD-1 cells in comparison to their parental counterparts (Fig. 7G).

## Discussion

This study showed that dose escalation treatment of 5-FU produced 5-FU desensitized CRC cells characterized by an increased tolerance to 5-FU compared to their parental counterparts. In accordance, de Angelis et al. observed more cell death in parental HCT-116 cells compared to previously desensitized cells upon exposure of 770 μM of 5-FU for 24 h [31]. Additionally, out of the three cell contexts, DLD-1 cells showed the highest proliferation rate in the presence of 5-FU in culture. Hence why they were desensitized with a maximum dose of 10 μM. 5-FU resistant DLD-1 cells were reported to have an activated PI3K/AKT signaling, together with an upregulation of the silent mating type information regulation 2 homolog 1 (Sirt1) [32]. Sirt1 is a histone deacetylase involved in carcinogenesis and chemoresistance. Therefore, these findings could account for the increased tolerance to 5-FU observed in the DLD-1 cells in comparison to the HCT-116 and Caco-2 cells.

In addition, the 5-FU desensitized CRC cells displayed several morphological features that the parental cells did not present. This could be another indication of acquiring 5-FU resistance. These included the presence of large, multinucleated cells together with an increased number of vacuoles. Indeed, Sousa-Squiavinato et al. observed that 5-FU resistant HCT-116 cells have a larger cell volume with lamellipodium-like membrane projections, stress fibers, and decreased cell-to-cell contacts [33]. Changes in cell morphology were similarly observed in other chemoresistant cells from various tumor types. For example, Zhang et al. observed a more spindle-shaped morphology and multiple pseudopodia in 5-FU resistant breast cancer cells [34]. Such alterations were associated with increased aggressiveness and poor prognosis [35–37]. Additionally, certain morphological characteristics have been associated with G1 cell cycle arrest, allowing the cancer cells to escape the effects of chemotherapeutic agents that primarily act during the S-phase of the cell cycle [36]. Although these cells appear to be ‘senescent-like’ under therapeutic stress, they still have the capability to re-enter the cell cycle, self-renew, and initiate tumors, once the drug is no longer present [36].

Subsequently, we investigated whether the acquisition of 5-FU desensitization was associated with increased metastatic potential in the three CRC cell lines. Indeed, dose-escalation 5-FU treatment reduced the migration and invasion abilities of the CRC cells. We then validated the observed reduction in metastatic potential of the cells by examining the protein expression of the key EMT markers E-cadherin, vimentin, and Snail. Moreover, this analysis would provide insights into whether EMT was among the mechanisms contributing to the observed increase in drug tolerance in the 5-FU desensitized cells. In this study, the reduction of Snail and vimentin in the desensitized cells showed that 5-FU desensitization did not induce EMT. Therefore, in agreement with the wound healing and invasion assays, dose-escalation 5-FU treatment reduced the aggressiveness of the CRC cells. However, many studies examining the EMT potential of 5-FU resistant CRC cells reported the opposite effect. For instance, 5-FU resistant HT-29 and HCT-116 cells displayed enhanced motility and invasive capabilities after 48 h, when compared to parental cells [14,33]. Moreover, 5-FU resistant HCT-8 cells were characterized by the up-regulation of vimentin protein levels [38]. That being said, these studies employed a different treatment strategy than the one used in this study, characterized by longer periods of recovery in drug-free medium (for example, between 30–45 days) [33]. In the absence of therapeutic stress, the surviving cells have time to recover and re-enter the cell cycle. Nevertheless, the daughter cells might not have identical gene expression patterns as their parent (5-FU-resistant) cells since the daughter cells have not been exposed to the drug [26]. This makes it difficult to study potential mechanisms associated with drug treatment and resistance and shows how the drug treatment strategy influences the nature of the results obtained. Additionally, the observed decrease in EMT potential in the 5-FU desensitized cells can be attributed to cell cycle arrest, aligning with the observed morphological characteristics in Fig. 2.

This study also looked at the effect of the continuous, dose-escalation 5-FU treatment approach on the protein expression of EHMT2, PRMT5, and SETD7/9. This gave insight into whether changes in the expression of these PMTs were associated with acquired 5-FU desensitization in CRC. In all 5-FU desensitized cells, the average protein expression of EHMT2 was lower than in the parental cells. Likewise, average PRMT5 levels were lower in drug-desensitized cells. This is once again in contrast with other studies on drug resistance. For instance, Olaparib-resistant ovarian adenocarcinoma cells and 5-FU resistant gastric cancer tissue both showed an increase in EHMT2 protein expression [39,40]. In addition, PRMT5 was found to be implicated in 5-FU resistance in CRC cells (HCT-116 and SW480 cell lines) *in vitro* and *in vivo* [41]. One exception in our results was found in one of the biological repeats of the 5-FU desensitized Caco-2 cells. Here, the protein levels of both EHMT2 and PRMT5 were higher than the parental cells. This lack of reproducibility highlights the intra- and inter-tumor heterogeneity characteristic of cancer. In the same tumor, there may be many different drug-resistant sub-clones that behave differently and thus the same cell line desensitized with the same chemotherapeutic drug can generate a diverse range of resistant cancer cells [26]. This was exemplified by Tegze et al., where the authors generated 29 different chemoresistant cell groups from MCF-7 and MDA-MB-231 breast cancer cells desensitized with long-term exposure to doxorubicin and paclitaxel [42].

On a similar note, a lack of reproducibility was also observed in SETD7/9 levels of the 5-FU desensitized Caco-2 and DLD-1 cells. The loss of SETD7/9 in some of these repeats may have supported the increased tolerance to 5-FU in the desensitized cells. It was reported that overexpression of SETD7/9 decreased the proliferation and migration of untreated HCT-116 and SW480 colon cells [43]. Additionally, SETD7/9 was shown to suppress histone deacetylase-6 (HDAC6)-mediated activation of the ERK signaling cascade, showing that SETD7/9 has an anti-tumor role in colon cancer [43]. However, this study did not look at the role of SETD7/9 in the CRC cells in the presence of chemotherapeutic agents [43]. As described earlier, it has been reported that SETD7/9 has also been associated with chemoresistance in certain cancers [22]. This shows that the expression and role of particular PMTs like SETD7/9 may vary across different cancers at various stages. Thus, it is important to study these changes in a context specific manner.

**Figure S1:**
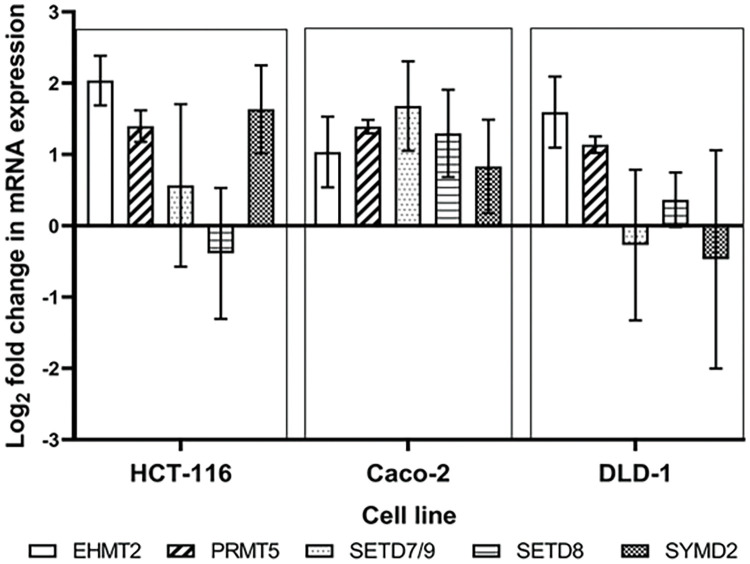
Dose escalation exposure of 5-FU promoted the dysregulation of PMT gene expression as shown by the log_2_ gene expression changes of EHMT2, PRMT5, SETD7/9, SETD8 and SYMD2 between the parental and 5-FU desensitized cells.

Furthermore, along with EHMT2, PRMT5, and SETD7/9, the PKMTs SET and MYND domain containing 2 (SYMD2) and SET domain 8 (SETD8) were also examined at an mRNA level through RT-qPCR (Suppl. Fig. S1). The expression of SETD8 was particularly high in 5-FU desensitized Caco-2 cells in contrast to their parental counterparts. The gene expression of SETD8 was also upregulated in the desensitized DLD-1 cells but downregulated in the desensitized HCT-116 cells. In contrast, SYMD2 was upregulated in the desensitized HCT-116 and Caco-2 cells but downregulated in the DLD-1 cells. The roles of SETD8 and SYMD2 in chemoresistance have been reported by various groups. For example, melphalan resistance in multiple myeloma, cisplatin resistance in cervical cancer, and docetaxel resistance in hepatocellular carcinoma were all associated with SETD8 [44–46]. Similarly, SYMD2 enhanced oxaliplatin resistance in SW620 CRC cells by inducing the expression of drug efflux transporters [47]. SYMD2 was also shown to enhance the drug sensitivity of 5-FU, doxorubicin, docetaxel, and cisplatin in renal cell carcinoma cells [48]. In addition, in this study, the gene expression of EHMT2, PRMT5, and SETD7/9 was up-regulated in all 5-FU desensitized cells. For the most part, this did not align with the observed protein expression analysis. The large variation in the fold mRNA expression between the biological repeats of the 5-FU desensitized cells also made it difficult to compare these findings with the protein expression data. Considering that the protein level carries out most biochemistry within the cell, and that numerous molecular processes affect the production of proteins, in this case, the protein analysis results were given priority.

Dysregulation in protein methylation has been linked to both carcinogenesis and drug resistance since it affects the expression of cancer-related genes [49]. For instance, the loss of trimethylation of histone 4 lysine 20 (H4K20) in primary tumor tissue and cancer cell lines has been linked to a poor prognosis in CRC [50]. This study showed that dose escalation 5-FU treatment stimulated a reduction in a number of methylated residues coupled with an increase of other methylations, which could contribute to 5-FU resistance. Furthermore, distinct methylation expression patterns were observed in each desensitized cell line, potentially attributed to variations in cellular mutations between the three cell contexts. It was expected that with the observed reduction of most PMTs in the 5-FU desensitized cells, an overall decrease in protein methylation would also be observed. However, it is predicted that there are over 100 PKMTs in the human genome, suggesting that a number of PMTs, other than the ones investigated brought about lysine methylations observed here [51]. Other PRMTs could have also been involved in the arginine methylation changes including PRMT9 which like PRMT5 is a type II PRMT and is thus responsible for the generation of SDMA residues [51]. Similarly, type I PRMTs including PRMT-1, 2, 3, 4, 6, and 8 are important for the generation of ADMA residues while PRMT7 is a type III PRMT, important for the mono-methylation of the terminal nitrogen on arginine residues [51,52]. Additionally, the hypermethylated proteins in the 5-FU desensitized CRC cells might also trigger a negative feedback loop that reduces the protein expression of the PMTs [26]. This would explain the observed decrease in the protein levels of the PMTs coupled with the increase in protein methylation observed in the drug-desensitized CRC cells [26]. Accordingly, cells are able to control the amount of methylated products. It is also important to look into the role of protein demethylases such as Lysine-specific histone demethylase 1A (KDM1A/LSD1) and Lysine demethylase 4B (KDM4B) in relation to 5-FU resistance since they mediate the removal of methyl groups and are thus also essential in regulating protein methylation in the cell [50].

**Figure S2:**
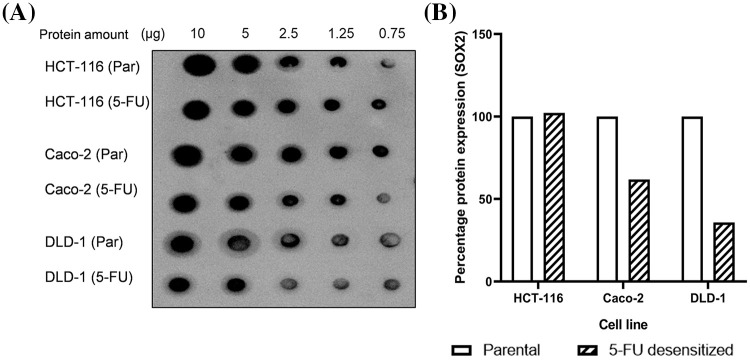
Dose escalation exposure of 5-FU did not enhance the cancer stem cell phenotype of CRC cells. (A) Dot Blot showing the expression of SOX2 in parental and 5-FU desensitized HCT-116, Caco-2 and DLD-1 cells at different protein amounts. (B) The quantification of % protein expression of SOX2 in parental and 5-FU desensitized cells across one technical and biological repeat at 2.5 µg.

Many studies report the role of the CSC marker SRY-box 2 (SOX2) not only in CSC formation but also in cancer cell proliferation, migration, invasion, and drug resistance [53]. Particularly, SOX2 expression was reported to be highly expressed in CRC cell lines (HCT-116, HT-29, Caco-2, DLD-1, LoVo, and SW480 cell lines) [54]. Furthermore, the silencing of SOX2 in SW620 CRC cells enhanced the cells’ sensitivity to 5-FU when compared to control cells [55]. For this reason, since the expected changes in EMT and PMT expression were not observed, we additionally explored if dose escalation 5-FU treatment promoted the development of stem cell characteristics in CRC cells. Using a dot blot, we measured the levels of SOX2 in parental and 5-FU desensitized HCT-116, Caco-2, and DLD-1 cells (Suppl. Fig. S2). SOX2 was observed in all parental and 5-FU desensitized cells, thus it might have influenced the carcinogenesis of the CRC cells. Nonetheless, apart from the desensitized HCT-116 cells which presented only a marginal increase in SOX2 levels (by 2%), SOX2 expression decreased in the 5-FU desensitized Caco-2 and DLD-1 cells. This shows that in this study, dose escalation treatment of 5-FU did not promote the acquisition of stem cell characteristics in CRC cells.

This study is limited by the substantial variation observed in the investigation of protein expression for the three PMTs in the 5-FU desensitized cells. This variability is also evident between the three CRC cell lines even though they are all colon adenocarcinoma cells. Although this allows for the opportunity to explore inherent variations between biological repeats, the highly context specific role of PMT expression and protein methylation provides a challenge to elucidate their role in cancer progression. Furthermore, the high-level laboratory model of drug resistance employed in this study is less clinically relevant compared to other drug treatment strategies that involve a pulsed treatment approach with lower drug doses and longer periods of time in drug-free medium. However, the treatment strategy adopted in this study is distinguished by stable levels of resistance, making it more suitable to study potential mechanisms of drug resistance. Additionally, it would have been ideal to consider other EMT markers in our investigation to get a more comprehensive overview of the effect of 5-FU desensitization in CRC. Although, we have tested more than one antibody for each of our EMT marker targets and the results were the same, the sharpness of the bands was of varying clarity and hence we only included the results from one biological repeat.

In agreement with previous reports, this study also sheds light on the significance of combining anti-cancer therapeutics with different modes of action to prevent the formation of drug-resistant clones. Additionally, the treatment strategy should also take into account the intra- and inter-tumor variability in gene and protein expression of cancer in order to optimize tumor response. For example, for tumors with overexpressed PRMT5, PRMT5 inhibitors can be investigated in combination with common chemotherapeutic agents to overcome chemoresistance. Indeed, the combination of the PRMT5 inhibitor, JNJ-64619178, and trametinib in glioblastoma displayed an increase in the number of apoptotic cells and number of cells in G1 cell cycle arrest than either individual therapy alone [56]. Thus, health care providers should also consider assessing the expression of PMTs when determining combinational drug regimens for individual cancer patients. Moreover, Liu et al. highlight how the use of methylation editing technologies can alter histone methylation marks to change the expression of target genes [23]. Thus, gene regulation can be an attractive therapeutic strategy that targets specific cancer cell populations with long-lasting effects.

In conclusion, this study showed that continuous, dose-escalation treatment of 5-FU in CRC cells generated 5-FU desensitized cancer cells that seemed to be less aggressive than the parental cells, characterized by reduced migratory and invasive abilities, reduced expression of EMT markers and reduced expression of EHMT2, PRMT5, and SETD7/9 together with changes in their protein methylation profile. However, drug-sensitized or chemoresistant cells can still retain the ability to initiate tumors *in vivo* once the treatment is stopped for an extended period of time [36]. Therefore, it makes sense in future studies to replicate this investigation on the 5-FU desensitized cells after an extended period of recovery in a drug-free medium, before making an informed decision whether such PMTs should be used as biomarkers in CRC.

## Data Availability

Derived data supporting the findings of this study can be made available from the corresponding author upon request.
